# High-resolution melting analysis for rapid and sensitive MYD88 screening in chronic lymphocytic leukemia

**DOI:** 10.3892/ol.2019.10582

**Published:** 2019-07-05

**Authors:** Min Jiang, Jie Li, Jun Zhou, Chao Xing, Jing-Jing Xu, Feng Guo

Oncol Lett 18: 814-821, 2019; DOI: 10.3892/ol.2019.10342

Subsequently to the publication of this article, the authors have realized that the article contained several errors that were not corrected. First, an error was introduced into Fig. 1; essentially, the mutation of T>C at position 794 of the nucleotide sequence of the wild-type *MYD88* plasmid was not portrayed correctly (as cytosine) in panel (B). In addition, the sequencing result in the lowest panel of Fig. 4 shows the wild-type sequence, and therefore the nucleotide at position 794 should have been presented as T, not as C. Corrected versions of Figs. 1 and 4 are shown opposite.

Otherwise, textual errors remained in the paper. Some textual errors were featured in the subsection of the Materials and methods entitled “*Sensitivity evaluation by HRM assay and direct sequencing*”, in the final paragraph in the right-hand column of p. 816. The sentence commencing 6 lines from the bottom of the page should have read as: “The melting curve obtained using 1% **negative control** template clearly differed from the **positive** template.” (“negative control” instead of “mutated”, and “positive” instead of “negative”). The subsequent sentence should have read as: “However, the mutation was detectable by direct sequencing when the **negative control template** frequency was >10% (Fig. 4).” (“negative control template” instead of “mutant”). Finally, some errors remained uncorrected in the legend for Fig. 4 on p. 819. The first sentence in the legend should have read as follows: “Direct sequencing results of **negative control template** at serial dilutions (100, 50, 20, 10, 5, 1 and 0%) with **positive** control.” (“negative control template” instead of “*MYD88* p.L265P mutations”, and “positive” instead of “negative”).

The authors and the Editor apologize to the readership of the Journal for any inconvenience caused.

## Figures and Tables

**Figure 1.**
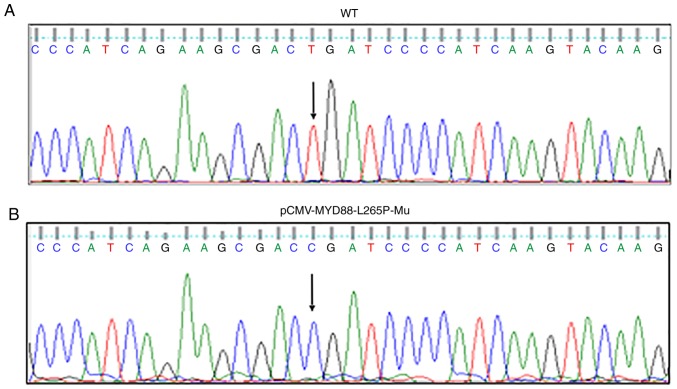
Sequencing result of two plasmids. (A) Sequence of the WT *MYD88* plasmid. There is no mutation at position 794 (arrow). (B) Sequence of the mutant *MYD88* plasmid pCMV-MYD88-L265P-Mu. There is a mutation at position 794 T>C (arrow). *MYD88*, myeloid differentiation primary response 88; WT, wild-type; Mu, mutated.

**Figure 4.**
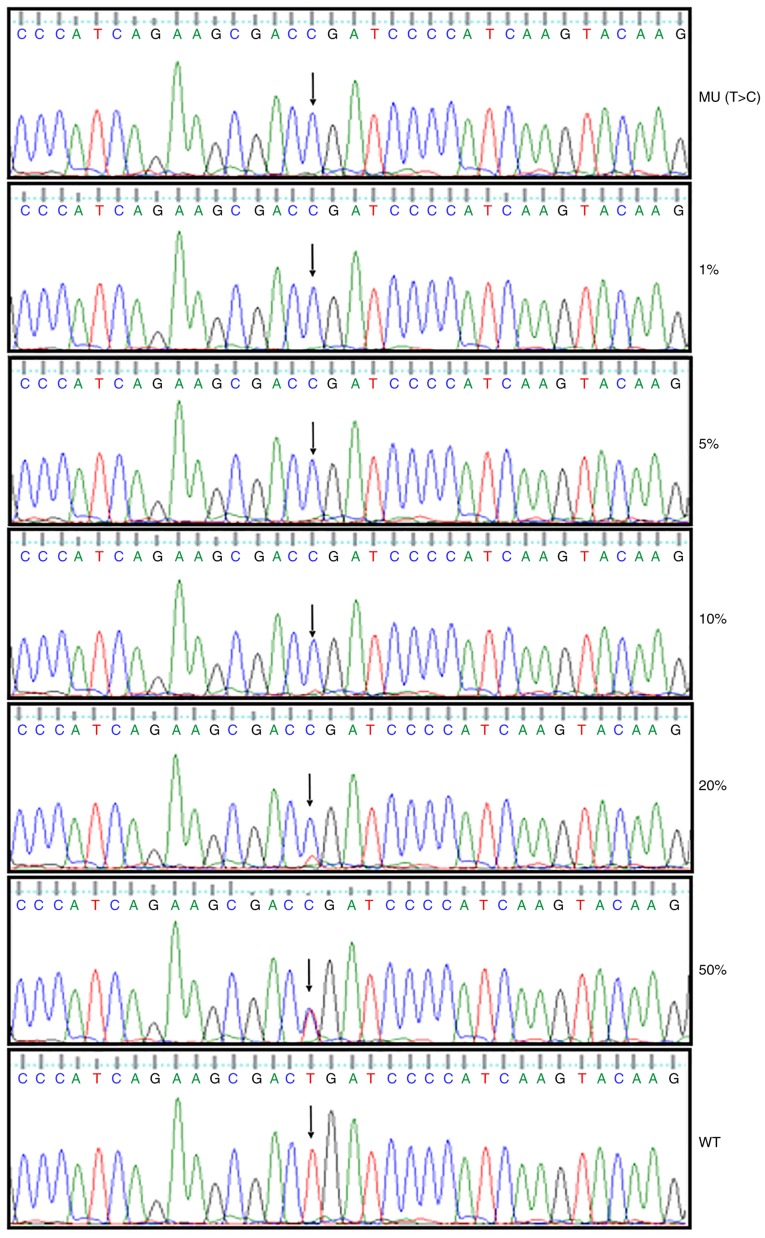
Validation and sensitivity testing for direct sequencing. Direct sequencing results of negative control template at serial dilutions (100, 50, 20, 10, 5, 1 and 0%) with positive control. The sensitivity for direct sequencing was indicated to be 10%. Arrows indicate the mutation site. WT, wild-type; MU, mutated.

